# Aging Increases Susceptibility to High Fat Diet-Induced Metabolic Syndrome in C57BL/6 Mice: Improvement in Glycemic and Lipid Profile after Antioxidant Therapy

**DOI:** 10.1155/2016/1987960

**Published:** 2016-02-18

**Authors:** Valéria Nunes-Souza, Cheila Juliana César-Gomes, Lucas José Sá Da Fonseca, Glaucevane Da Silva Guedes, Salete Smaniotto, Luíza Antas Rabelo

**Affiliations:** ^1^Laboratório de Reatividade Cardiovascular (LRC), Núcleo de Síndrome Metabólica, Instituto de Ciências Biológicas e da Saúde (ICBS), Universidade Federal de Alagoas (UFAL), Avenida Lourival Melo Mota, s/n, Cidade Universitária, 57072-900 Maceió, AL, Brazil; ^2^National Institute of Science and Technology in Nano-Biopharmaceutics (N-BIOFAR), Avenida Antônio Carlos, s/n, Pampulha, 31270-901 Belo Horizonte, MG, Brazil; ^3^Departamento de Fisiologia e Farmacologia, Centro de Ciências Biológicas (CCB), Universidade Federal de Pernambuco (UFPE), Avenida Professor Moraes Rego 1235, Cidade Universitária, 50670-901 Recife, PE, Brazil; ^4^Faculdade de Nutrição (FANUT), Universidade Federal de Alagoas (UFAL), Avenida Lourival Melo Mota, s/n, Cidade Universitária, 57072-900 Maceió, AL, Brazil; ^5^Laboratório de Biologia Celular, Instituto de Ciências Biológicas e da Saúde (ICBS), Universidade Federal de Alagoas (UFAL), Avenida Lourival Melo Mota, s/n, Cidade Universitária, 57072-900 Maceió, AL, Brazil; ^6^Max Delbrück Center for Molecular Medicine, Robert-Rössle-Straße 10, 13125 Berlin, Germany

## Abstract

Nonalcoholic fatty liver disease (NAFLD) has been considered a novel component of the metabolic syndrome (MetS), with the oxidative stress participating in its progression. This study aimed to evaluate the metabolic profile in young and old mice with MetS, and the effects of apocynin and tempol on glycemic and lipid parameters. Young and old C57BL/6 mice with high fat diet- (HFD-) induced MetS received apocynin and tempol 50 mg·kg^−1^/day in their drinking water for 10 weeks. After HFD, the young group showed elevated fasting glucose, worsened lipid profile in plasma, steatosis, and hepatic lipid peroxidation. Nevertheless, the old group presented significant increase in fasting insulin levels, insulin resistance, plasma and hepatic lipid peroxidation, and pronounced steatosis. The hepatic superoxide dismutase and catalase activity did not differ between the groups. Tempol and apocynin seemed to prevent hepatic lipid deposition in both groups. Furthermore, apocynin improved glucose tolerance and insulin sensitivity in old mice. In summary, old mice are more susceptible to HFD-induced metabolic changes than their young counterparts. Also, the antioxidant therapy improved insulin sensitivity and glucose tolerance, and in addition, apocynin seemed to prevent the HFD-induced hepatic fat deposition, suggesting an important role of oxidative stress in the induction of NAFLD.

## 1. Introduction

Nonalcoholic fatty liver disease (NAFLD), characterized by fatty infiltration of the liver, has been considered a novel component of the metabolic syndrome (MetS) [1–4], the latter being a multifactorial condition that gathers various cardiovascular risk factors and metabolic disorders [2, 3].

NAFLD encompasses various degrees of liver damage, ranging from simple steatosis to a complex and more dangerous presentation, steatohepatitis [5], and it is often accompanied by oxidative stress [6], a condition of imbalance in the prooxidant/antioxidant balance in favor of prooxidants [7].

The liver is a central organ for the maintenance of systemic lipid and glucose homeostasis and, due to this fact, it is susceptible to oxidative stress damage [8, 9]. The latter plays a critical role in the progression of metabolic disorders, including insulin resistance, one of the conditions which characterize the NAFLD [4]. In addition, oxidative stress may induce several pathophysiological events in the liver, such as detrimental effects on hepatocytes by damaging DNA, lipids, and proteins, ultimately leading to disruptions in cellular homeostasis [2, 10].

Nicotinamide adenine dinucleotide phosphate oxidase (NAD(P)H oxidase) is an enzyme involved in the progression of the disorders related to the MetS, such as liver injury [11], hyperglycemia [12], increased oxidative stress in diabetes and obesity [2, 13], hypertension [14], and impaired endothelium-dependent dilation [15]. Also, that enzyme appears to play an essential role in a higher insulin-stimulated hydrogen peroxide (H_2_O_2_) generation in skeletal muscle [16].

Although NAD(P)H oxidase is the major source of reactive oxygen species (ROS) production [13, 17], the antioxidant enzymes superoxide dismutase (SOD), catalase (CAT), and glutathione peroxidase (GPx) have a critical importance in the metabolism of ROS [7]. SOD is responsible for dismuting the superoxide anion (^∙^O_2_^−^) into H_2_O_2_, which, in turn, is removed by CAT and GPx, finally being converted into water and O_2_ [7]. In this scenario, these enzymes exert an important protective function against ROS, which are increased with age [18, 19] and in high fat diet- (HFD-) induced obesity, as well as in MetS and NAFLD animal models [1, 4, 9, 20].

Even though several studies have been conducted to assess the metabolic effects of the consumption of HFD in animal models [16, 21, 22], little is known about its effect concomitant with antioxidant therapy in old age and in the MetS. Based upon this scenario, we hypothesized that aged mice are more sensitive to HFD-induced metabolic changes than younger mice and that the concomitant antioxidant therapy is effective in reducing the negative metabolic impacts from HFD consumption. In order to test this hypothesis, we assessed the metabolic profile in young and old C57BL6 mice presenting with MetS and sought to investigate the preventive effects of apocynin, a NAD(P)H oxidase inhibitor, and tempol, a SOD mimetic, on HFD-induced metabolic changes in those animals.

## 2. Materials and Methods

### 2.1. Animals and Experimental Procedures

C57BL/6 male young and old mice used in this study were purchased from Federal University of Alagoas at 6–8 weeks and 54–56 weeks of age, respectively. During all the experimental period, mice were kept on a 12-hour light/dark cycle and fed and hydrated* ad libitum*, with controlled humidity and temperature. All procedures were carried out in accordance with the criteria outlined in the Guide for the Care and Use of Laboratory Animals (NIH publication 86-23, 1996) and approved by the Institutional Ethics Committee of the Federal University of Alagoas (Protocol 005208/2010-46).

Young (Y) and old (O) mice were divided into two groups and fed different diets during 14 weeks: high fat diet, which was developed by our group, with the other nutrient contents in accordance with the American Institute of Nutrition's recommendation (AIN-93) [23, 24] (HF-Y and HF-O) (58.4% kcal lipids from lard; 26.6% kcal carbohydrates, i.e., sucrose; 15% kcal protein, i.e., casein) or standard chow diet (CT-Y and CT-O) (11.8% kcal lipids, 62.6% kcal carbohydrates, and 25.6% kcal protein). After 4 weeks of HFD (the required period for the glycemic alterations to appear, a time interval previously detected in other experimental groups conducted by our laboratory, with blood glucose levels assessed weekly—Accu-Chek* Performa*, Roche®, São Paulo, Brazil), the HF-Y and HF-O groups were divided into three subgroups according to their treatment: (1) positive control that continued receiving only HFD and normal water (HF-Y and HF-O); (2) HFD plus oral treatment with tempol (50 mg·kg^−1^/day, dissolved in drinking water) (HFT-Y and HFT-O); (3) HFD plus oral treatment with apocynin (50 mg·kg^−1^/day, dissolved in drinking water) (HFA-Y and HFA-O). The exact duration of the tempol and apocynin interventions was 10 weeks. The dosages of tempol and apocynin were recalculated weekly, taking into account the mean body weight recorded every week and the liquid consumption of the groups.

### 2.2. Intraperitoneal Glucose Tolerance Test (GTT) and Insulin Tolerance Test (ITT)

At the end of treatment, fasting blood glucose was assessed (12 h fast) using a glucometer (Accu-Chek* Performa*, Roche, São Paulo, Brazil). Next, 2 g glucose/kg of body weight was intraperitoneally injected and blood from the tail vein was collected again at 15, 30, 60, 90, and 120 minutes for measuring glucose levels. Two days later, baseline blood glucose was measured in fed state. Sequentially, insulin (0.75 units/kg of body weight; Humulin®, Eli Lilly, Indianapolis, IN) was intraperitoneally administered and tail blood samples were obtained at 15, 30, and 60 minutes to obtain blood glucose levels.

### 2.3. Euthanasia and Organ Collection

After intraperitoneal anesthesia using a xylazine/ketamine solution (10/110 mg·kg^−1^), the cephalocaudal length and abdominal circumference were measured (cm) and the animals were sacrificed by exsanguination through cardiac puncture of the right ventricle. Blood was collected and centrifuged and plasma was separated and stored at −80°C until biochemical analyses were performed. Liver, adipose tissues, heart, gastrocnemius muscle, and intestine were carefully perfused, removed, and weighed. Next, one piece of the right hepatic lobe was selected for histological analysis, and two more pieces (~100 mg) were immediately snap-frozen in dry ice and stored at −80°C for assessing lipid profile and the activities of antioxidant enzymes. Similarly, adipose tissues (epididymal, perirenal, and mesenteric) were stored at −80°C for further analysis. White adipose tissue (WAT) index was calculated following the formula: WAT index (%) = [(epididymal fat (g) + perirenal fat (g))/(body weight (g)) *∗* 100] [25]. The relative weight (%) of adipose tissue was calculated as follows: relative weight (%) = [(adipose tissue (g)/body weight (g)) *∗* 100]. Liver, gastrocnemius, heart, and intestine mass were normalized by tibial length and expressed as g/mm of tibial length. Finally, right tibias were also removed and had their lengths measured.

### 2.4. Plasma Lipid Analysis

Total cholesterol, triglyceride, and free fatty acids (FFA) levels in plasma were assayed using commercial kits (enzymatic colorimetric assay; Labtest®, Lagoa Santa, Brazil, and Wako Chemicals GmbH®, Neuss, Germany), following the manufacturer's instructions with adaptations for microplate (Thermo Scientific®, Software 2.4 Multiskan Spectrum, Vantaa, Finland). VLDL was calculated as follows: VLDL = triglyceride/5 [26].

### 2.5. Insulin Measurement and Markers of Insulin Resistance

Insulin levels were quantified using an ELISA assay (Millipore®, Schwalbach, Germany) according to the manufacturer's instructions. The product of fasting triglycerides and glucose (TyG index) was calculated following the formula: TyG index = Ln [fasting triglyceride (mg·dL^−1^) × fasting glucose (mg·dL^−1^)/2] [27, 28]. The homeostasis model assessment (HOMA index) was calculated as follows: HOMA index = [fasting glucose (mg·dL^−1^) × fasting insulin (*µ*U·mL^−1^)/405] [29].

### 2.6. Evaluation of Liver Injury

Commercial kits (Labtest, Lagoa Santa, Brazil) adapted for microplate reading (Thermo Scientific, Software 2.4 Multiskan Spectrum, Vantaa, Finland) were used for evaluating in plasma the degree of liver injury and function by measuring the enzymatic concentrations of alanine aminotransferase (ALT), aspartate aminotransferase (AST), alkaline phosphatase (ALP), *γ*-glutamyltransferase (*γ*-GT), and lactate dehydrogenase (LDH). Levels of total protein [30] and albumin were also determined.

### 2.7. Liver Histological Analysis

In order to investigate liver structure and the occurrence of steatosis, small hepatic fragments were fixed in 10% buffered formaldehyde, embedded in paraffin, sectioned at 4–6 *µ*m, and stained with hematoxylin and eosin (H&E). The sections from each animal were histologically examined in light microscope (Olympus®, Tokyo, Japan) and photographed (Kodak digital camera, Eastman Kodak Company, Rochester, NY, USA).

### 2.8. Liver Lipids Analysis

Total hepatic lipids were extracted from liver samples according to a gravimetric standard method [31], followed by measurement of cholesterol and triglyceride levels using commercial kits (enzymatic colorimetric assays; Labtest, Lagoa Santa, Brazil) according to the manufacturer's instructions, with adaptations for microplate (Thermo Scientific, Software 2.4 Multiskan Spectrum, Vantaa, Finland), and VLDL as described [26].

### 2.9. Protein Extraction from Liver Samples

From each mouse, a piece of frozen liver was homogenized in a RIPA lysis buffer (Cell Signaling®, Beverly, MA, USA) plus protease and phosphatase inhibitor cocktails (Roche, Mannheim, Germany). Following the extraction, total protein concentration was quantified by the Bradford method [30] and the samples were stored at −80°C for subsequent measurements.

### 2.10. Measurement of Lipid Peroxidation

Thiobarbituric acid reactive substances (TBARS), mainly malondialdehyde (MDA), are widely used as a marker of lipid peroxidation. Such quantification was performed according to Ohkawa et al. [32] with some adaptations. Briefly, 100 *µ*L of hepatic homogenate samples or 100 *µ*L of plasma was individually mixed with 10 *µ*L of butylated hydroxytoluene (final concentration = 1 mmol·L^−1^) and 1250 *µ*L of thiobarbituric acid (1.3%; Sigma Aldrich®, Seelze, Germany). Then, samples were heated at 100°C during 60 minutes, being next incubated in ice bath (~4°C) for 10 minutes to stop the reaction. Sequentially, samples were centrifuged at 1600 g per 15 minutes at 4°C. In order to remove the interfering absorbance, for each sample, the value of the absorbance at 600 nm was subtracted from the value read at 532 nm. Finally, values were normalized for total protein concentration and expressed as nM·mg protein^−1^ [30].

### 2.11. Superoxide Dismutase (SOD) and Catalase (CAT) Activities

Total SOD activity was assessed in 20 *µ*L of liver homogenate with a commercial colorimetric kit (Sigma Aldrich, Seelze, Germany) following the manufacturer's instructions and read in a microplate reader (Thermo Scientific, Software 2.4 Multiskan Spectrum, Vantaa, Finland) at 450 nm. The obtained values were normalized by total protein concentration in the liver [30] and expressed as IU·protein^−1^, mg of hepatic protein. Catalase activity was measured according to Xu et al. [33], being expressed as *µ*mol/min/mL·protein^−1^, mg [30].

### 2.12. Statistical Analysis

The data are expressed as mean ± standard error of the mean (SEM), and *p* < 0.05 was considered statistically significant. Analyses were performed using the one- or two-way ANOVA, when applicable (followed by* Bonferroni's post hoc* test) (Graph pad Prism® 5.0, San Diego, CA, USA).

## 3. Results

### 3.1. General Parameters, Fat Deposition, and Lipid Profile in Mice with MetS and Treated with Antioxidant Therapy

The measurements of food intake, animal growth, abdominal circumference, and lipids and glucose metabolism, variables which can be changed during the consumption of a HFD, were done to evaluate the possible effects of HFD and of the antioxidant treatment with apocynin and tempol in mice at different ages.

The data showed that the food intake, regardless of age, was statistically lower in HFD-fed groups (HF-Y and HF-O; Table 1). However, the energy consumed did not differ between groups, since the HFD displays a caloric content greater than the chow diet, thus normalizing the caloric intake (data not shown). Besides, abdominal circumference was increased in HF-O compared to CT, without differences between the young groups. With respect to the influence of HFD on animal growth, no statistical difference was observed in tibial and cephalocaudal length between young and old groups. Neither tempol nor apocynin had effect on any of these aforementioned parameters during HFD intake in both groups (Table 1).

**Figure 1:**
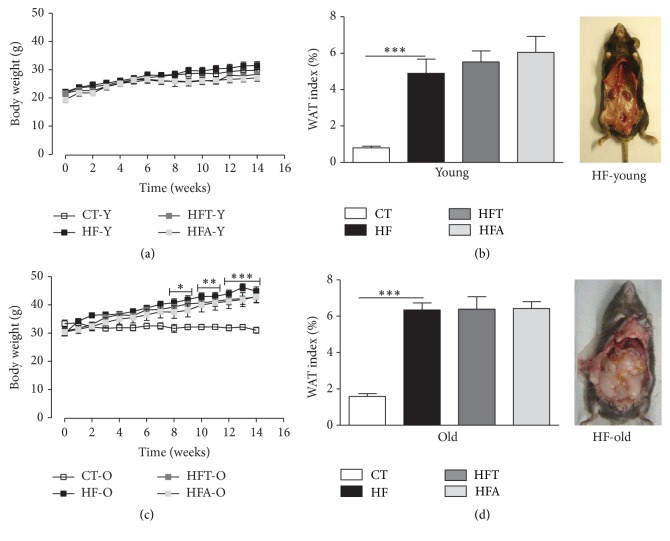
Body weight and WAT index. Monitoring of body weight (g) during the weeks before and after pharmacological intervention with tempol (50 mg·kg^−1^) and apocynin (50 mg·kg^−1^) in young (a) and old (c) C57BL/6 mice. WAT index (%) and representative pictures of the HF diet animals after pharmacological intervention in young (b) and old (d) C57BL/6 mice. CT = control group; HF = high fat diet group; HFT = high fat diet + tempol group; HFA = high fat diet + apocynin group. Each point of the graph represents the mean ± SEM. ANOVA (two-way) with* Bonferroni's post hoc* test: ^*∗*^ *p* < 0.05; ^*∗∗*^ *p* < 0.01; ^*∗∗∗*^ *p* < 0.001, CT versus HF. Each bar graph represents the mean ± SEM. ANOVA (one-way) with* Bonferroni's post hoc* test: ^*∗∗∗*^ *p* < 0.001, CT versus HF (*n* = 5-6).

Although no significant difference in body weight was observed between the young groups during the HFD consumption (Figure 1(a)), the WAT index (Figure 1(b)) and relative epididymal, perirenal, and mesenteric fat weight (Table 1) were significantly higher. However, in one of the old groups (HF-O), a significant increase was observed in body weight compared with CT-O from the 8th week (*p* < 0.05) until the end of treatment (*p* < 0.001) (Figure 1(c)), indicating that aging enhances the metabolic effects associated with HFD consumption. The WAT index (Figure 1(d)) and the relative epididymal, perirenal, and mesenteric fat weight (Table 1) were also significantly higher in old animals after HFD consumption. In both young and old groups, apocynin and tempol had no effects on body weight, WAT index, and relative adipose tissue weight during HFD intake when compared to the respective control (HF-Y and HF-O, resp.).

**Figure 2:**
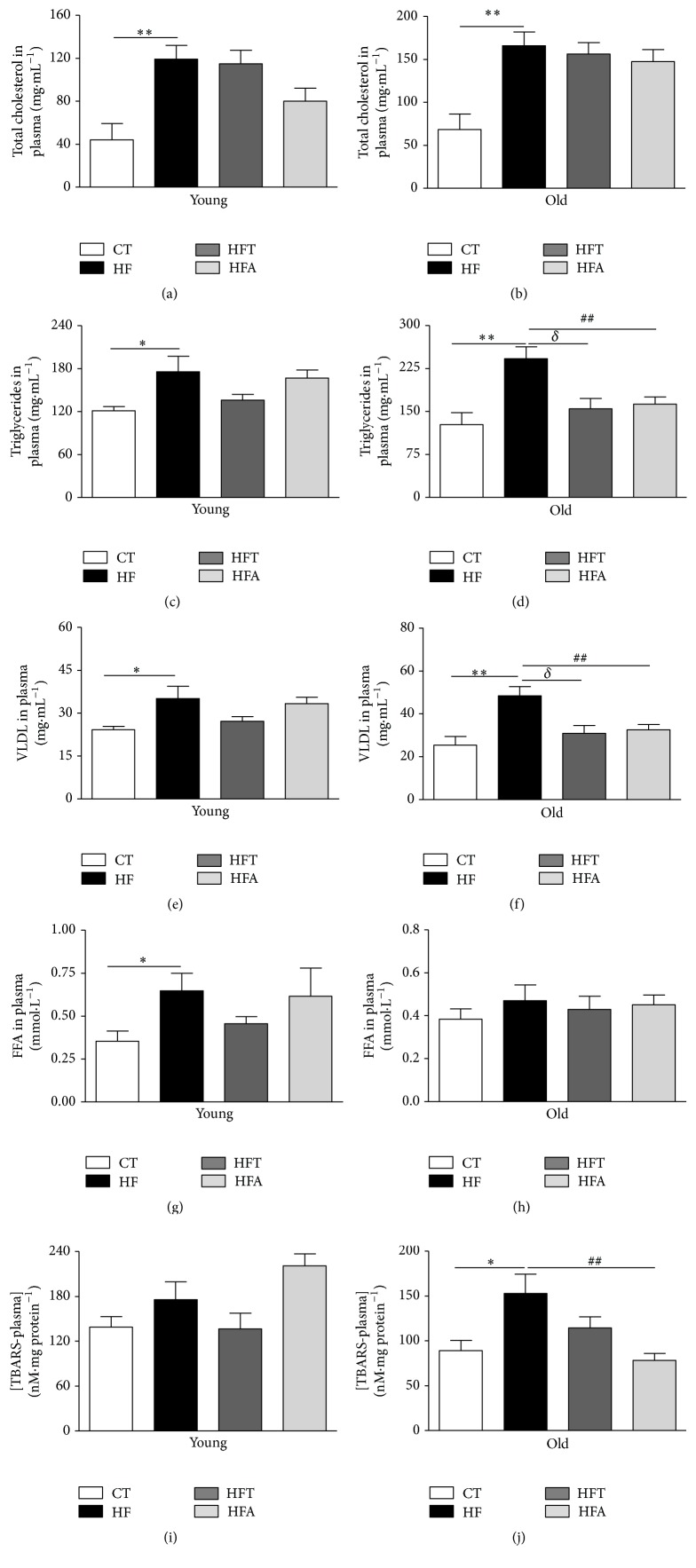
Lipid profile in plasma. Total cholesterol in plasma (mg·mL^−1^) in young (a) and old (b) C57BL/6 mice. Triglycerides in plasma (mg·mL^−1^) in young (c) and old (d) C57BL/6 mice. Very-low-density lipoprotein (VLDL) in plasma (mg·mL^−1^) in young (e) and old (f) C57BL/6 mice. Free fatty acids (FFA) in plasma (mmol·L^−1^) in young (g) and old (h) C57BL/6 mice. Lipid peroxidation in plasma ([TBARS] nM·mg protein^−1^) in young (i) and old (j) C57BL/6 mice. CT = control group; HF = high fat diet group; HFT = high fat diet + tempol group; HFA = high fat diet + apocynin group. Each bar graph represents the mean ± SEM. ANOVA (one-way) with* Bonferroni's post hoc* test: ^*∗*^ *p* < 0.05; ^*∗∗*^ *p* < 0.01, CT versus HF; ^*δ*^ *p* < 0.05, HFT versus HF; ^##^ *p* < 0.01, HFA versus HF (*n* = 5-6).

The HFD did not change skeletal muscle mass (gastrocnemius mass) in young and old groups (Table 1). On the other hand, the intestinal mass was significantly decreased in both young and old groups that received HFD. In addition, the heart mass was significantly lower in the HF-Y but not in HF-O (Table 1), suggesting that induction of muscle loss by consumption of HFD is more pronounced in the younger compared to older age. HF-Y mice, although not presenting an increase in body weight, showed a markedly elevated concentration of total cholesterol, triglycerides, VLDL, and FFA in plasma compared with CT-Y (Figures 2(a), 2(c), 2(e), and 2(g), resp.). Similarly, HF-O mice presented elevated total cholesterol, triglycerides, and VLDL levels but not FFA compared to the CT-O (Figures 2(b), 2(d), 2(f), and 2(h), resp.). Lipid peroxidation was not different between the young groups, but the old HFD group presented significant increase in this parameter, which was decreased after apocynin treatment (Figures 2(i) and 2(j)). In the old group, both tempol and apocynin were also able to decrease the levels of triglycerides and VLDL in plasma (Figures 2(d) and 2(f)).

### 3.2. Glycemic Profile in Mice with MetS and Treated with Antioxidant Therapy

**Figure 3:**
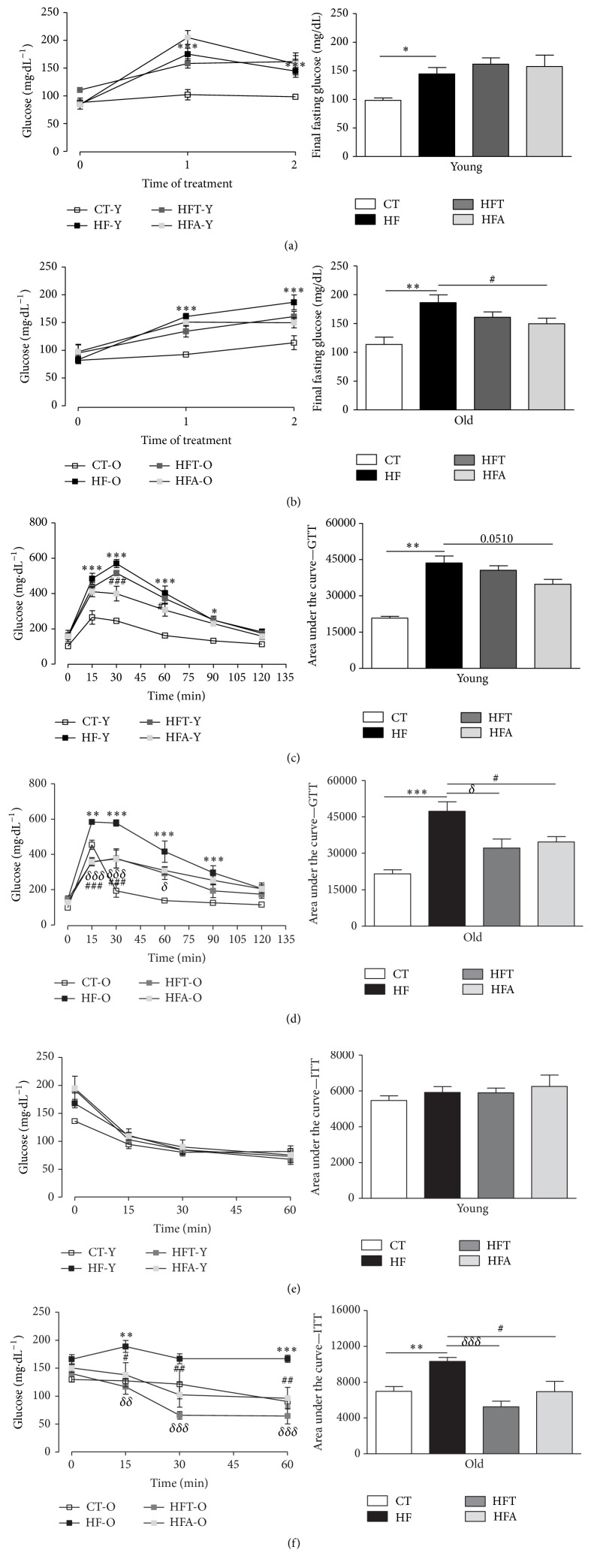
Glycemic profile. Fasting glucose (mg·dL^−1^) at three different points (basal fasting glucose: Time 0; beginning of pharmacological intervention with tempol (50 mg·kg^−1^) and apocynin (50 mg·kg^−1^): Time 1; and the end of pharmacological intervention: Time 2) and end time of fasting glucose in young (a) and old (b) C57BL/6 mice. Evaluation of glucose tolerance and Area under the curve of the test (AUC) in young (c) and old (d) C57BL/6 mice. Evaluation of insulin sensitivity and area under the curve of the test (AUC) in young (e) and old (f) C57BL/6 mice. CT = control group; HF = high fat diet group; HFT = high fat diet + tempol group; HFA = high fat diet + apocynin group. Each point of the graph represents the mean ± SEM. ANOVA (two-way) with* Bonferroni's post hoc* test: ^*∗*^ *p* < 0.05; ^*∗∗*^ *p* < 0.01; ^*∗∗∗*^ *p* < 0.001, CT versus HF; ^*δδ*^ *p* < 0.01; ^*δδδ*^ *p* < 0.001, HFT versus HF; ^#^ *p* < 0.05; ^##^ *p* < 0.01; ^###^ *p* < 0.001, HFA versus HF. Each bar graph represents the mean ± SEM. ANOVA (one-way) with* Bonferroni's post hoc* test: ^*∗*^ *p* < 0.05; ^*∗∗*^ *p* < 0.01; ^*∗∗∗*^ *p* < 0.001, CT versus HF; ^*δ*^ *p* < 0.05; ^*δδδ*^ *p* < 0.001, HFT versus HF; ^#^ *p* < 0.05, HFA versus HF (*n* = 5-6).

As shown in Figure 3, the basal levels of glucose (Time 0) in young and old mice were not different. However, after receiving HFD during 4 weeks (Time 1), these groups (HF-Y and HF-O) presented significantly higher fasting glucose compared to their respective CT groups (CT-Y and CT-O). At this point, we started the pharmacological intervention with tempol and apocynin during 10 weeks. At the end of treatment (Time 2), regardless of age, no statistical difference in glucose concentration was observed for tempol groups (HFT-Y, HFT-O) compared to their respective HFD groups (HF-Y and HF-O). Nevertheless, apocynin significantly decreased the final fasting glucose in old animals with MetS, but not in the young ones (Figures 3(a) and 3(b)).

HF-Y and HF-O mice presented increased glucose intolerance (Figures 3(c) and 3(d)). This effect was evident by the higher time-dependent glucose concentration at 15, 30, 60, and 90 min after the intraperitoneal injection of glucose, as well as by the significant increase in the AUC in the HF-Y and HF-O groups compared with the correspondent CT group. Apocynin, a NAD(P)H oxidase inhibitor, seems to prevent the occurrence of such effects in both young and old mice with MetS. On the other hand, tempol, a SOD mimetic, was shown to be capable of reducing the occurrence of the aforementioned changes only in old mice.

Interestingly, the young mice (HF-Y) did not show decreased insulin sensitivity after HFD consumption, a fact observed in old mice (HF-O) during the ITT, and when calculating the AUC from these data (Figures 3(e) and 3(f)). Time-dependent glucose concentration in ITT and the AUC decreased in the HFT-O and HFA-O compared with the HF-O group, indicating that both apocynin and tempol improved insulin sensitivity in old mice. In addition, we measured the fasting insulin concentrations in plasma, with no differences observed in young groups after HFD, but with a significant increase in the old group. Additionally, in the treated groups, neither tempol nor apocynin had effects on that parameter in the old group. However, in the young group, both treatments decreased the insulin levels compared to the HFD group (Table 2). Analysis of the insulin resistance markers, TyG index and HOMA index, showed that only in old animals these parameters were increased after HFD consumption, with both treatments, tempol and apocynin, being able to significantly decrease the TyG index in old animals (Table 2).

### 3.3. Function, Lipid Profile, and Redox State in Liver Samples of Mice with MetS and Treated with Antioxidant Therapy

In order to evaluate the effect of the improved glycemic profile on liver metabolism after pharmacological intervention, we assessed markers of liver injury and function, fat accumulation, and hepatic redox balance.

**Figure 4:**
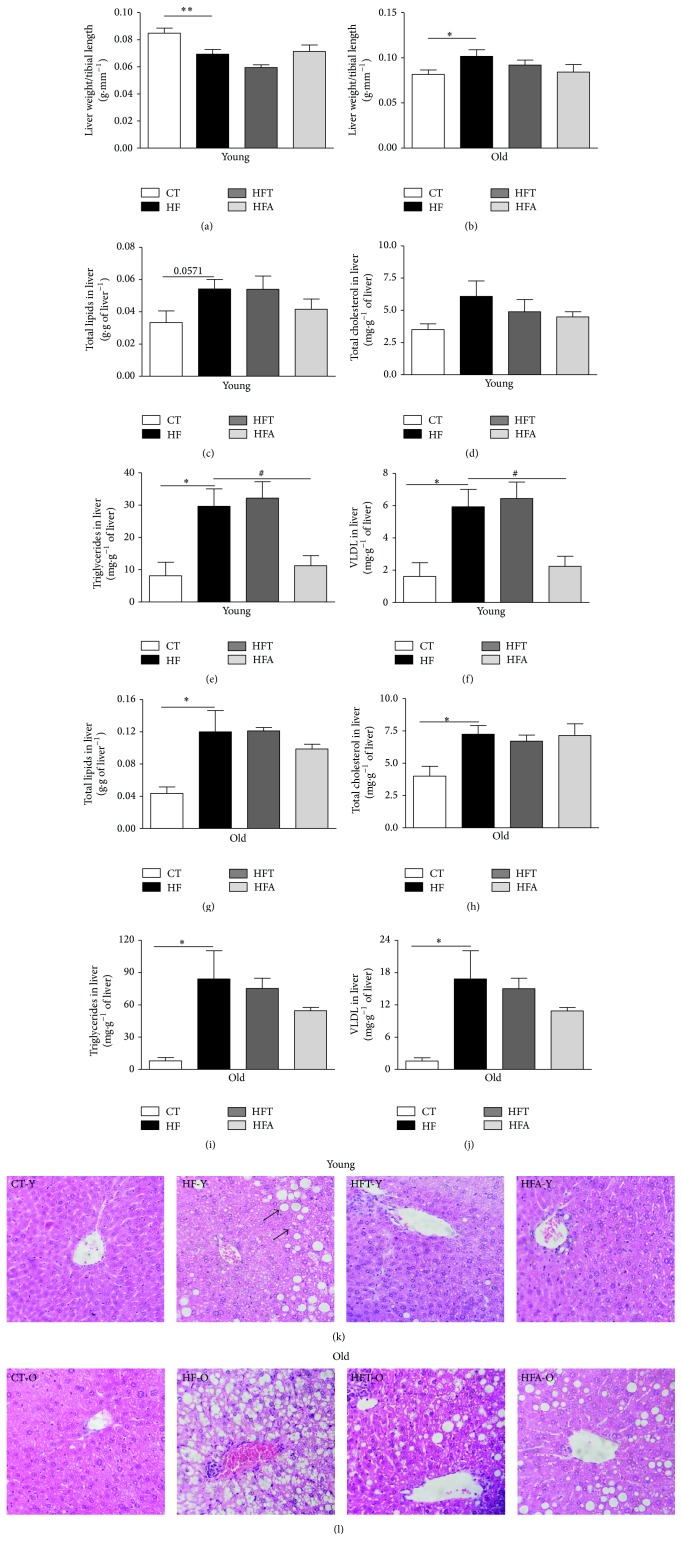
Lipid profile in liver. Liver weight/tibial length (g·mm^−1^) in young (a) and old (b) C57BL/6 mice. Total lipids in liver (g·g^−1^ of liver) in young (c) and old (g) C57BL/6 mice. Total cholesterol in liver (mg·g^−1^ of liver) in young (d) and old (h) C57BL/6 mice. Triglycerides in liver (mg·g^−1^ of liver) in young (e) and old (i) C57BL/6 mice. Very-low-density lipoprotein (VLDL) in liver (mg·g^−1^ of liver) in young (f) and old (j) C57BL/6 mice. Liver histological section from young (k) and old (l) C57BL/6 mice stained with hematoxylin and eosin (400x magnification). The photomicrographs represent the main histological findings in the experimental groups. CT = control group; HF = high fat diet group; HFT = high fat diet + tempol group; HFA = high fat diet + apocynin group. The arrows indicate the fat vesicles (micro and macro). Each bar graph represents the mean ± SEM. ANOVA (one-way) with* Bonferroni's post hoc* test: ^*∗*^ *p* < 0.05; ^*∗∗*^ *p* < 0.01, CT versus HF; ^#^ *p* < 0.05, HFA versus HF (*n* = 5-6).

The liver weight in young mice that consumed HFD was significantly lower compared to that observed in animals fed standard chow diet (Figure 4(a)). On the other hand, the HF-O showed a significant increase in liver weight (Figure 4(b)). The analysis of plasma markers of hepatocellular injury (ALT, AST, and LDH), canalicular enzymes (ALP, *γ*-GT), and markers of liver function (albumin and total proteins) is shown in Table 2. In both young and old mice, AST and ALT levels were increased in HF group compared to CT group, indicating the elevated hepatic injury in these animals after the HFD consumption. The treatment with tempol decreased the levels of AST and ALT in plasma of young animals, but not in the old ones. Additionally, the other hepatic markers evaluated were not found to be different between the groups.

The quantification of total lipids and fractions (total cholesterol, triglycerides, and VLDL) showed an increase after HFD consumption in both young and old animals (Figures 4(c)–4(j)). No significant changes were observed in the concentration of all the parameters listed above in groups treated with tempol. However, apocynin treatment was able to decrease the levels of triglycerides and VLDL in liver of young animals with MetS (Figures 4(e) and 4(f)).

Histological assessment from HF-Y and HF-O liver samples presented a pattern of NAFLD, characterized by fatty infiltration (Figures 4(k) and 4(l)). Furthermore, HFD groups (HF-Y and HF-O) showed hepatocellular disorganization and inflammatory zones. Qualitatively, the groups that received tempol (HFT-Y and HFT-O) showed a decrease in hepatic fat accumulation, specifically in macro vesicular steatosis, compared to the respective HFD group. A similar condition was observed for the groups treated with apocynin (HFA-Y and HFA-O) (Figures 4(k) and 4(l)).

**Figure 5:**
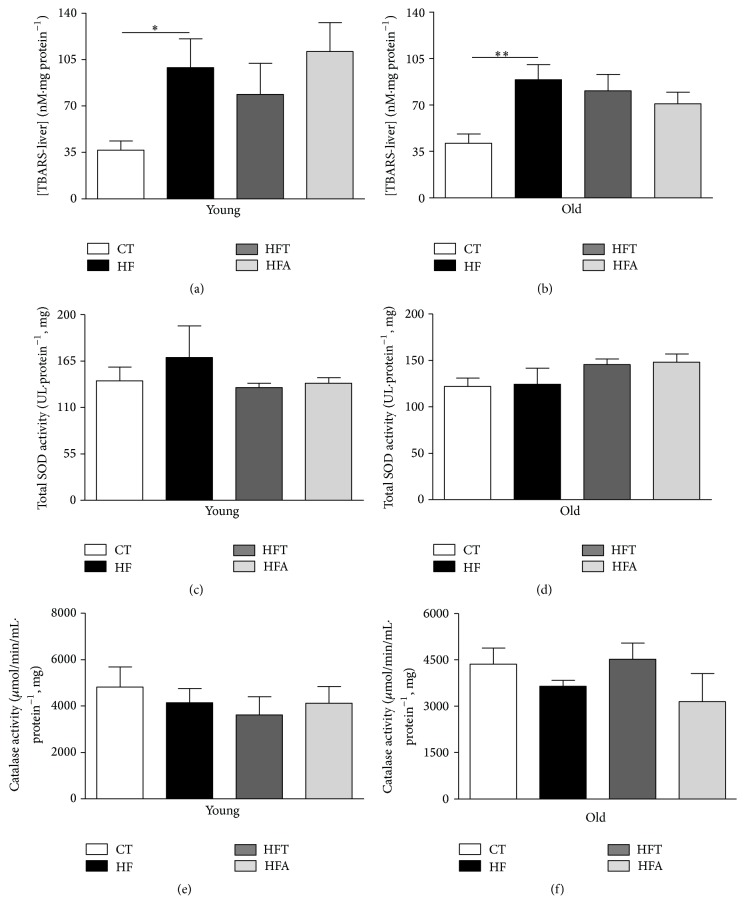
Markers of redox state in liver. Hepatic lipid peroxidation ([TBARS] nM·mg protein^−1^) in young (a) and old (b) C57BL/6 mice. Total superoxide dismutase activity (SOD: UI·protein^−1^, mg) in young (c) and old (d) C57BL/6 mice. Catalase activity (*µ*mol/min/mL·protein^−1^, mg) in young C57BL/6 (e) and old mice (f). Each bar graph represents the mean ± SEM. ANOVA (one-way) with* Bonferroni's post hoc* test: ^*∗*^ *p* < 0.05; ^*∗∗*^ *p* < 0.01, CT versus HF (*n* = 5-6).

The evaluation of the hepatic redox state was based on the quantification of malondialdehyde as a lipid peroxidation measure (Figures 5(a) and 5(b)), as well as the activities of SOD (Figures 5(c) and 5(d)) and CAT (Figures 5(e) and 5(f)) in liver homogenates. Consumption of HFD increased lipid peroxidation, without changes in SOD and CAT activities in liver samples from young (HF-Y) and old (HF-O) mice compared to their respective controls (CT-Y and CT-O). Furthermore, these parameters were not altered after the treatment with tempol and apocynin for both young and old mice.

## 4. Discussion

The major findings of this work are the markedly increased weight gain in aged mice fed a HFD compared to younger ones, as well as the observation that the concomitant intake of HFD and the treatment with antioxidant agents, here represented by apocynin and tempol, improve insulin sensitivity and glucose tolerance in aged mice. Our work also demonstrated that HFD intake induces metabolic alterations similar to those observed in humans presenting with MetS and that NAFLD associated with dietary changes indeed constitute another risk factor for the MetS, the advanced age being an important contributing factor for augmenting such alterations, mainly obesity and hepatic fat deposition [3].

Aging is characterized by a decline in biological functions, with direct influence of lifestyle [34–36]. The data presented herein showed that HFD intake for 14 weeks did not induce body weight gain in young mice (20–22 weeks old at the end of the dietary intervention). Nevertheless, this period was effective in inducing a significant increase in body weight in aged animals. However, young animals presented a greater loss of heart and liver tissue when ingesting HFD, which could compensate the HFD-induced obesity expected in this MetS model. Taken together, these data also evidence that aging and HFD synergistically contribute to accentuating the decline in body capacity to metabolize fat, leading to a greater accumulation of visceral adiposity.

Despite the fact that the HFD-fed young mice did not present significant increase in body weight, other metabolic parameters were shown to be considerably augmented due to the HFD consumption, similar to the observations for the old ones, reflecting, in both groups, the onset of MetS [37–39]. Among these metabolic alterations, increased fasting glucose, impaired glucose tolerance, increased lipid profile in plasma, and greater abdominal and hepatic fat deposition, associated with increased AST and ALT plasma levels, could be numbered. However, HFD-induced insulin resistance, augmentation in TyG and HOMA indexes, and the increase in plasma insulin concentration were only observed in aged animals.

It is already known that the main contributing factor in the aging process and the early development of metabolic diseases is represented by the oxidative stress [34], with the latter enhancing the metabolic and vascular effects evoked by HFD [39, 40]. In this sense, the referred couple of factors, advanced age and dietary changes, might contribute to increase the production of ROS which, in turn, directly contribute to the onset and/or maintenance of glucose intolerance and insulin resistance [13, 41]. Notwithstanding, after treatment with tempol and apocynin, concomitant with the HFD intake, a significant improvement in these parameters related to glucose metabolism was observed in aged mice, in addition to the decrease in triglycerides and VLDL in plasma. More interestingly, apocynin also decreased these parameters in liver, corroborating the findings of improved insulin sensitivity, as the augmented levels of these markers in the liver are associated with insulin resistance. Together, these data suggest that the increase in NAD(P)H oxidase activity and the concentration of ^∙^O_2_^−^ may be involved in the mechanisms associated with the regulation of the glycemic and lipid profile, with the presence of oxidative stress being possible in this dietary model for MetS.

Tempol acts as a scavenger of ^∙^O_2_^−^ [41, 42]. Therefore, it is plausible to assume that the use of this pharmacological agent in the aged group in the current study may have contributed to the reduction of ^∙^O_2_^−^ and, thus, to the improvement in glucose tolerance and insulin sensitivity. In accordance with this statement, Banday and colleagues [41] found that tempol ameliorates insulin sensitivity by promoting a reduction in oxidative stress in obese Zucker rats, this way improving insulin-mediated glucose uptake.

Apocynin, through its antioxidant action and/or its inhibitory effect on the NAD(P)H oxidase enzymatic complex, probably contributed to the observed metabolic improvement at least partially by the reduced participation of ROS [13, 20], ultimately suggesting that the increased activity of the NAD(P)H oxidase enzymatic complex may play an essential role in mechanisms related to the impaired glycemic profile observed in the present work.

In line with these observations, Espinosa and colleagues [16] showed that an increase in NAD(P)H oxidase 2 expression in skeletal muscle appears to be involved in the augmented H_2_O_2_ release upon insulin stimulation in insulin-resistant mice [16]. Furthermore, the same study found that, after treatment with apocynin for 8 weeks, HFD-fed mice showed improved glucose tolerance and decreased fasting insulin concentration. In addition, another experimental work showed that the treatment with apocynin for 5 weeks in HFD-fed mice was effective in improving glucose tolerance and insulin sensitivity [9]. These studies, in part, corroborate our findings concerning the antioxidant treatment and glucose metabolism.

Advanced age also favored significant accumulation of abdominal fat, a situation which implies a negative impact on glycemic control [43]. Furthermore, the significant hepatic fat deposition was more pronounced in aged animals, as observed by the intense presence of micro and macro fat vacuoles. Concerning this issue, the relation between age and the degree of obesity and fat deposition was observed, due to the consumption of HFD for 14 weeks. However, after treatment with tempol and apocynin, the qualitative analysis from liver histology showed a less accentuated presence of fat vacuoles, quantitatively confirmed in HFA-O group by a decrease in triglycerides and VLDL. Additionally, tempol reduced the hepatic damage caused by HFD, as observed by the reduction of AST and ALT, both markers of liver injury. These data suggest that the antioxidant activity of apocynin and the scavenger activity of tempol on ^∙^O_2_^−^ seem to reduce the hepatic oxidation of fatty acids during the chronic treatment concomitant with the consumption of HFD, preventing liver injury, and that the increased susceptibility of HFD-induced MetS in old mice could be due to the increase of redox imbalance during the aging process. Corroborating the histological findings of the present work, several studies showed the HFD-induced hepatic fat accumulation [3, 40].

Still, while assessing the liver histology, the presence of inflammatory agglomerates suggests a direct participation of hepatic fat accumulation in the elevated index of lipid peroxidation observed in this organ, once the inflammatory milieu evoked by fat deposition may contribute to augmenting the local production of ROS. Taken together, these data suggest that HFD induced a state of steatohepatitis, a condition which characterizes the NAFLD [8], and that local oxidative stress may be present and contribute in a direct manner to the progression of NAFLD as well [6, 44]. Such occurrence could be more likely due to the excessive production of ROS than by a decrease and/or deficiency of antioxidant capacity, since the activity of antioxidant enzymes SOD and CAT did not significantly differ between HFD and chow diet groups.

The products generated in the process of lipid peroxidation act as reactive agents, causing cell damage, which amplifies the oxidative stress effects. Moreover, they induce the production of inflammatory cytokines and subsequent fibrosis, via activation of hepatic stellate cells [45]. Additionally, there exists a correlation between plasma and hepatic oxidative stress and clinical and histological findings in patients presenting with NAFLD [44]. Accordingly, these observations reassert the contribution of oxidative stress in the progression of NAFLD in humans and, under this aspect, our study points to the possible participation of the altered oxidative state, arising from HFD consumption, in the onset of steatohepatitis.

In this context, our results also indicate that the normal hepatic redox state may be altered, possibly by an increase in NAD(P)H oxidase activity and the elevated ^∙^O_2_^−^ production. Uchiyama and colleagues [46] suggest that oxidative stress enhances hepatic fat deposition due to the degradation of apolipoprotein B in the liver, indicating that such molecule is a target for ROS. Reinforcing these observations, such degradation process is caused by lipid peroxidation, as stated in a study by Pan and colleagues [25].

In this sense, our data suggest that increased hepatic lipid peroxidation in HFD animals may have directly contributed to inhibiting hepatic release of VLDL to the systemic milieu. In addition, different studies showed apocynin as an effective pharmacological agent in ameliorating hyperlipidemia, hepatic steatosis [2], and insulin resistance [9], besides reducing inflammatory factors such as leptin, interleukin-6, and TNF-*α* [47], thereby indicating a protective role in diseases related to the MetS and in the progression of NAFLD.

Concerning the effects of apocynin on the oxidative state, Meng and colleagues [9] showed that lipid peroxidation, both hepatic and in plasma, was significantly reduced after an oral treatment with such compound for 5 weeks. Furthermore, the authors observed an increase in SOD activity and a reduction in CAT activity in the liver, after treatment with apocynin. Paradoxically, our findings differ in part from those in the aforementioned study, since only in plasma, but not in liver, the lipid peroxidation was decreased after apocynin treatment in old mice. However, once the advanced age itself represents a factor which culminates with the emergence of redox imbalance [39], it is plausible to suggest that in the liver the difference between treated and untreated groups with antioxidant agents might have been masked by this factor.

In summary, our findings demonstrate that old mice are more susceptible to HFD-induced metabolic changes than young ones, with ROS generation presenting a close relationship with the progression of NAFLD. Furthermore, the improvement in glucose and lipid metabolism-related parameters and the attenuation of the progression of NAFLD in MetS mice, by using antioxidant agents, appears to be due to the ability of these pharmacological agents to inhibit the generation and accumulation of reactive species. Collectively, our results point to the cross talk in the triad HFD-aging-oxidative stress, once the HFD-induced metabolic derangements may be more pronounced with the advance of age and also potentially amenable to pharmacological interventions.

## Figures and Tables

**Table 1:** Intake and biometric parameters from young and old C57BL/6 mice after pharmacological intervention with tempol (50 mg·kg^−1^) and apocynin (50 mg·kg^−1^).

Intake and biometric parameters	Young mice				Old mice			
	CT-Y	HF-Y	HFT-Y	HFA-Y	CT-O	HF-O	HFT-O	HFA-O
Food intake (g/mouse/body weight)	0.15 ± 0.005	0.07 ± 0.003^a^	0.06 ± 0.003	0.07 ± 0.003	0.13 ± 0.006	0.05 ± 0.003^a^	0.06 ± 0.003	0.05 ± 0.004
Right tibia (cm)	1.62 ± 0.01	1.59 ± 0.01	1.59 ± 0.01	1.58 ± 0.02	1.66 ± 0.01	1.64 ± 0.01	1.64 ± 0.01	1.61 ± 0.01
Cephalocaudal length (cm)	17.56 ± 0.23	17.27 ± 0.19	16.88 ± 0.15	17.23 ± 0.16	18.39 ± 0.15	18.58 ± 0.13	18.49 ± 0.18	18.58 ± 0.30
Abdominal circumference (cm)	9.36 ± 0.40	9.47 ± 0.20	8.55 ± 0.05	8.23 ± 0.44	8.65 ± 0.16	10.48 ± 0.31^a^	10.26 ± 0.29	10.41 ± 0.38
Epididymal fat weight (%)	0.62 ± 0.07	3.36 ± 0.52^a^	3.83 ± 0.40	3.62 ± 0.84	1.09 ± 0.10	3.79 ± 0.26^a^	3.77 ± 0.38	3.72 ± 0.30
Perirenal fat weight (%)	0.18 ± 0.02	1.54 ± 0.26^a^	1.70 ± 0.20	1.38 ± 0.38	0.49 ± 0.06	2.55 ± 0.24^a^	2.54 ± 0.28	2.73 ± 0.23
Mesenteric fat weight (%)	1.55 ± 0.10	2.10 ± 0.3^a^	2.44 ± 0.21	2.49 ± 0.26	1.78 ± 0.09	3.25 ± 0.29^a^	3.82 ± 0.31	3.57 ± 0.23
Gastrocnemius (g)/tibial length (mm)	0.010 ± 0.00	0.009 ± 0.00	0.009 ± 0.00	0.01 ± 0.00	0.010 ± 0.00	0.01 ± 0.00	0.009 ± 0.00	0.01 ± 0.00
Heart (g)/tibial length (mm)	0.009 ± 0.00	0.008 ± 0.00^a^	0.007 ± 0.00^b^	0.008 ± 0.00	0.009 ± 0.00	0.009 ± 0.00	0.009 ± 0.00	0.01 ± 0.00
Intestine (g)/tibial length (mm)	0.073 ± 0.00	0.046 ± 0.00^a^	0.040 ± 0.00	0.053 ± 0.00	0.071 ± 0.00	0.050 ± 0.00^a^	0.046 ± 0.00	0.036 ± 0.00^c^

Y = young; O = old; CT = control group; HF = high fat diet group; HFT = high fat diet + tempol group; HFA = high fat diet + apocynin group. Values represent the mean ± SEM. ANOVA (one-way) with *Bonferroni's post hoc* test: ^a^ *p* < 0.05, HF versus CT; ^b^ *p* < 0.05, HFT versus HF; ^c^ *p* < 0.05, HFA versus HF. *n* = 5-6.

**Table 2:** Systemic glycemic and liver function markers from young and old C57BL/6 mice after pharmacological intervention with tempol (50 mg·kg^−1^) and apocynin (50 mg·kg^−1^).

Parameters	Young mice				Old mice			
	CT-Y	HF-Y	HFT-Y	HFA-Y	CT-O	HF-O	HFT-O	HFA-O
Insulin (ng·mL^−1^)	0.66 ± 0.21	0.56 ± 0.06	0.34 ± 0.04^b^	0.37 ± 0.02^c^	0.44 ± 0.06	0.59 ± 0.02^a^	0.57 ± 0.07	0.55 ± 0.04
Insulin resistance (TyG index)	8.77 ± 0.10	9.11 ± 0.29	9.29 ± 0.10	9.57 ± 0.22	8.81 ± 0.18	10.0 ± 0.13^a^	9.43 ± 0.15^b^	9.51 ± 0.10^c^
HOMA index	4.80 ± 1.71	3.32 ± 0.48	3.34 ± 0.53	3.42 ± 0.45	2.86 ± 0.51	6.56 ± 0.58^a^	5.76 ± 0.69	5.59 ± 0.63
AST (U·L^−1^)	44.30 ± 7.27	65.14 ± 3.14^a^	42.54 ± 6.06^b^	59.86 ± 5.63	15.80 ± 2.87	32.69 ± 3.86^a^	44.25 ± 3.11	42.16 ± 9.3
ALT (U·L^−1^)	9.56 ± 1.27	24.89 ± 0.72^a^	10.10 ± 1.17^b^	16.64 ± 5.07	6.11 ± 0.72	17.51 ± 2.13^a^	12.26 ± 4.06	17.92 ± 5.52
ALP (U·L^−1^)	3195 ± 17.61	3246 ± 68.51	3212 ± 55.59	3285 ± 50.97	3114 ± 36.28	3076 ± 24.97	3232 ± 80.17	3245 ± 74.60
*γ*−GT (U·L^−1^)	828.2 ± 32.80	977.8 ± 100.8	916.7 ± 31.91	1014 ± 66.68	756.7 ± 6.69	781.8 ± 16.85	891.0 ± 56.35	923.8 ± 58.82
LDH (U·L^−1^)	535.8 ± 21.36	593.2 ± 32.11	545.4 ± 5.63	643.6 ± 54.86	528.0 ± 6.5	521.9 ± 8.89	589.2 ± 25.28	562.8 ± 21.06
Albumin (mg·mL^−1^)	78.51 ± 0.66	78.48 ± 0.57	80.20 ± 1.05	78.59 ± 0.64	77.64 ± 0.80	77.69 ± 1.4	78.79 ± 0.9	78.69 ± 0.36
Total proteins (mg·mL^−1^)	5.125 ± 0.64	8.373 ± 1.63	8.502 ± 1.01	6.411 ± 1.11	4.687 ± 1.17	5.855 ± 1.18	8.910 ± 2.9	10.58 ± 1.38

Y = young; O = old; CT = control group; HF = high fat diet group; HFT = high fat diet + tempol group; HFA = high fat diet + apocynin group. Values represent the mean ± SEM. ANOVA (one-way) with *Bonferroni's post hoc* test: ^a^ *p* < 0.05, HF versus CT; ^b^ *p* < 0.05, HFT versus HF; ^c^ *p* < 0.05, HFA versus HF. *n* = 5-6. TyG: insulin resistance index; HOMA index: homeostasis model assessment; AST: aspartate aminotransferase; ALT: alanine aminotransferase; ALP: alkaline phosphatase; *γ*-GT: gamma glutamyltransferase; LDH: lactate dehydrogenase.
